# A Soft Coral-Derived Compound, 11-Dehydrosinulariolide, Induces G2/M Cell Cycle Arrest and Apoptosis in Small Cell Lung Cancer

**DOI:** 10.3390/md16120479

**Published:** 2018-11-30

**Authors:** Yu-Chao Lin, Jui-Hsin Su, Shih-Chao Lin, Chia-Che Chang, Te-Chun Hsia, Yu-Tang Tung, Chi-Chien Lin

**Affiliations:** 1 Graduate Institute of Clinical Medical Science, China Medical University, Taichung 404, Taiwan; D10001@mail.cmuh.org.tw; 2 Division of Pulmonary and Critical Care Medicine, Department of Internal Medicine, China Medical University Hospital, Taichung 404, Taiwan; derrick.hsia@msa.hinet.net; 3 Department of Respiratory Therapy, China Medical University, Taichung 404, Taiwan; 4 National Museum of Marine Biology and Aquarium, Pingtung 944, Taiwan; x2219@nmmba.gov.tw; 5 National Center for Biodefense and Infectious Diseases, School of Systems Biology, George Mason University, Manassas, VA 20110, USA; slin20@gmu.edu; 6 Institute of Biomedical Science, National Chung-Hsing University, Taichung 40227, Taiwan; chia_che@dragon.nchu.edu.tw; 7 Graduate Institute of Metabolism and Obesity Sciences, Taipei Medical University, Taipei 110, Taiwan; 8 Department of Medical Research, China Medical University Hospital, Taichung 404, Taiwan

**Keywords:** 11-dehydrosinulariolide, soft coral, small cell lung cancer, apoptosis, cell cycle arrest

## Abstract

11-Dehydrosinulariolide, an active compound that is isolated from the cultured soft coral *Sinularia flexibilis*, has been suggested to show anti-tumor biological characteristics according to previous studies. However, its potential effect on small cell lung cancer (SCLC) remains unknown. The present study investigates the underlying mechanism for the treatment of SCLC in vitro and in vivo. Cell viability was examined using the methyl-thiazol-diphenyl-tetrazolium (MTT) assay. Flow cytometry was applied to evaluate cell cycle distribution and apoptosis. The expression of proteins related to the cell cycle and apoptosis was analyzed by Western blot analysis. Additionally, an in vivo study was performed to determine the anti-SCLC effect on an H1688 subcutaneous tumor in a BALB/c nude mouse model. 11-Dehydrosinulariolide inhibited cell growth, triggered G2/M arrest and induced H1688 cell apoptosis in a dose- and time-dependent manner. Additionally, 11-dehydrosinulariolide caused the accumulation of p53 and Bax, accompanied by the activation of DNA damage-inducing kinases, including ataxia-telangiectasia mutated (ATM) and checkpoint kinase 2 (CHK2). Moreover, 11-dehydrosinulariolide increased the activity of caspase-3 and -7, suggesting that caspases are involved in 11-dehydrosinulariolide-induced apoptosis. 11-Dehydrosinulariolide also increased the level of tumor suppressor phosphatase and tensin homolog (PTEN) and inhibited the expression of phosphorylated Akt. In the in vivo study, the intraperitoneal injection of 11-dehydrosinulariolide at a dosage of 10 mg/kg significantly inhibited tumor growth compared with the control treatment. Together, the data indicate that 11-dehydrosinulariolide induces G (2)/M cell cycle arrest and apoptosis through various cellular processes, including the upregulation of p53 and Bax, activation of ATM and Chk2, activation of caspase-3 and -7, and accumulation of PTEN, leading to inhibition of the Akt pathway. These findings suggest that 11-dehydrosinulariolide might serve as a promising chemotherapy drug in the treatment of SCLC.

## 1. Introduction

Lung cancer is the most common cancer and exhibits the highest mortality rate among all cancer types in the world [1]. According to the specific histopathological features of the disease, the two major types of lung cancer are small cell lung cancer (SCLC) and nonsmall cell lung cancer (NSCLC). SCLC is the most aggressive form of lung cancer and accounts for 10%~15% of all lung cancers [2]. Clinically, SCLC grows rapidly and is more easily metastasized to other parts of the body. To reduce the high mortality rate, many researchers have focused on lung cancer prevention methods, as well as early detection and more effective treatments [3]. Although improvements have been made in lung cancer diagnosis and prevention, the effectiveness of the available treatment options remains limited. According to the American Cancer Society guidelines [4], chemotherapy is often part of SCLC treatment, and cisplatin, etoposide, carboplatin, and irinotecan are the most commonly used drugs. However, the current SCLC chemotherapy is not effective [5], and thus the potential mechanism underlying the development of lung cancer tumors has been sought to identify new treatment options.

Recently, several metabolites derived from marine organisms have become potential and valuable sources of anticancer drug candidates. 11-Dehydrosinulariolide, which is a cembranolide analog isolated from the cultured soft coral *Sinularia flexibilis*, showed several bioactivities [6,7,8]. 11-Dehydrosinulariolide could treat Parkinson’s disease through its anti-apoptotic and anti-inflammatory action via PI3K signaling [6]. 11-Dehydrosinulariolide has antiapoptotic and anti-inflammatory effects to improve functional recovery after spinal cord injury [7]. Recent reports have indicated that 11-dehydrosinulariolide exerts its antitumor effects by either caspase-dependent mitochondrial dysfunction-related or endoplasmic reticulum (ER) stress pathways in human oral squamous cell carcinoma cells Ca9-22 [8] and in human melanoma cells A2058 [9].

Although these findings demonstrate the anticancer activity of 11-dehydrosinulariolide, the underlying molecular mechanisms of the antitumor activity of 11-dehydrosinulariolide remain unclear. Additionally, there is no report on the antitumor effects of 11-dehydrosinulariolide against human small cell lung cancer (SCLC) cells. Hence, the aim of this study was to evaluate the effect of 11-dehydrosinulariolide on the antiproliferation of SCLC cells in vitro and in vivo and to investigate the potential molecular mechanisms. Particularly, we sought insights into the mechanism of action of 11-dehydrosinulariolide as well as its effects on cell proliferation, the cell cycle distribution, apoptosis, and expression levels of several cell cycle- and apoptosis-related proteins.

## 2. Results

### 2.1. 11-Dehydrosinulariolide Reduces H1688 and H146 Cell Viability In Vitro

**Figure 1:**
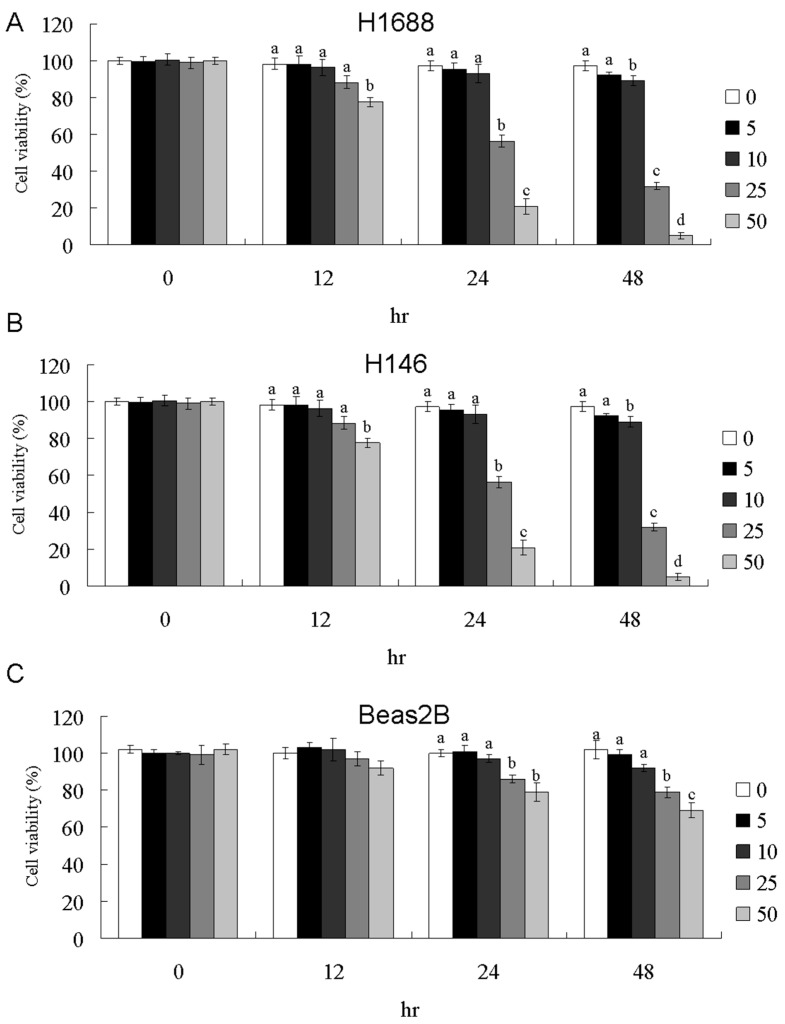

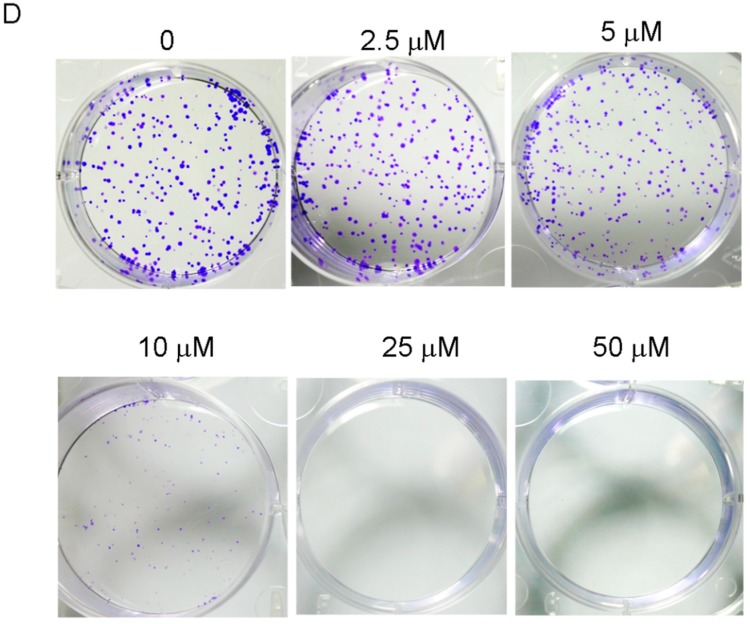

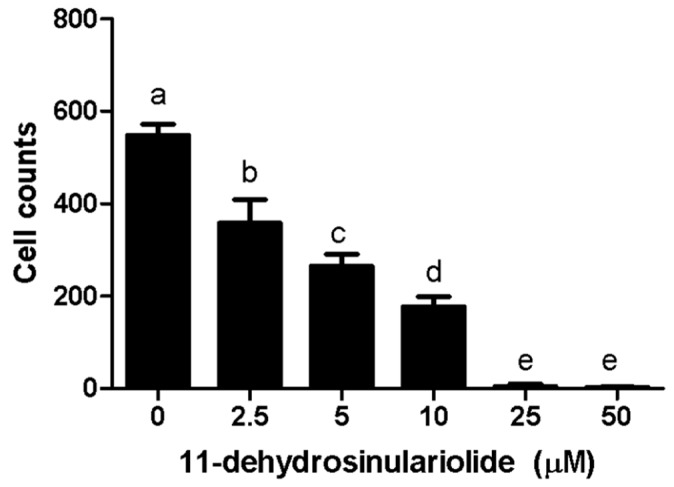
Effects of 11-dehydrosinulariolide on cell viability in H1688 (**A**), H146 (**B**) or Beas-2B (**C**) cells. The cells were treated with different concentrations (0, 5, 10, 25, and 50 μM) of 11-dehydrosinulariolide for 12, 24 and 48 h. The cell viability was measured using the MTT assay. Colony formation assay of H1688 (**D**) following treatment with 11-dehydrosinulariolide for 1 week. The data are presented as means ± SD from triplicate samples for each treatment.

Dose- and time-dependent changes in H1688 and H146 cell viability were determined using the MTT assay after incubation periods of 12, 24, and 48 h. As shown in Figure 1A,B, treatment with 11-dehydrosinulariolide caused significant antiproliferative effects on H1688 and H146 SCLC cells as indicated by dose- and time-dependent changes in cell viability, whereas 11-dehydrosinulariolide exhibited a moderate antiproliferative effect on human bronchial epithelial cells BEAS-2B (Figure 1C). The 50% growth inhibitory concentration (IC50) values of 11-dehydrosinulariolide after 12, 24 and 48 h of exposure, as calculated by the MTT assay, were as follows: >50, 29.8 ± 3.4, and 19.1 ± 2.4 μM, respectively, for H1688 cells; and >50, 43.5 ± 6.6, and 25.1 ± 2.6 μM, respectively, for H146 cells. In BEAS-2B cells, the IC50 was >50 μM even after 48 h of exposure. Similarly, colony formation assays showed dose-dependent inhibition of H1688 (Figure 1D) after 1-week treatment of colony formation by 11-dehydrosinulariolide, further confirming the cell growth inhibition effect of 11-dehydrosinulariolide. Furthermore, H1688 cells were more sensitive to 11-dehydrosinulariolide than H146 cells. Therefore, subsequent experiments were conducted using H1688 cells.

### 2.2. 11-Dehydrosinulariolide Induces Cell Cycle G2/M-Phase Arrest and Apoptosis in H1688 Cells

**Figure 2:**
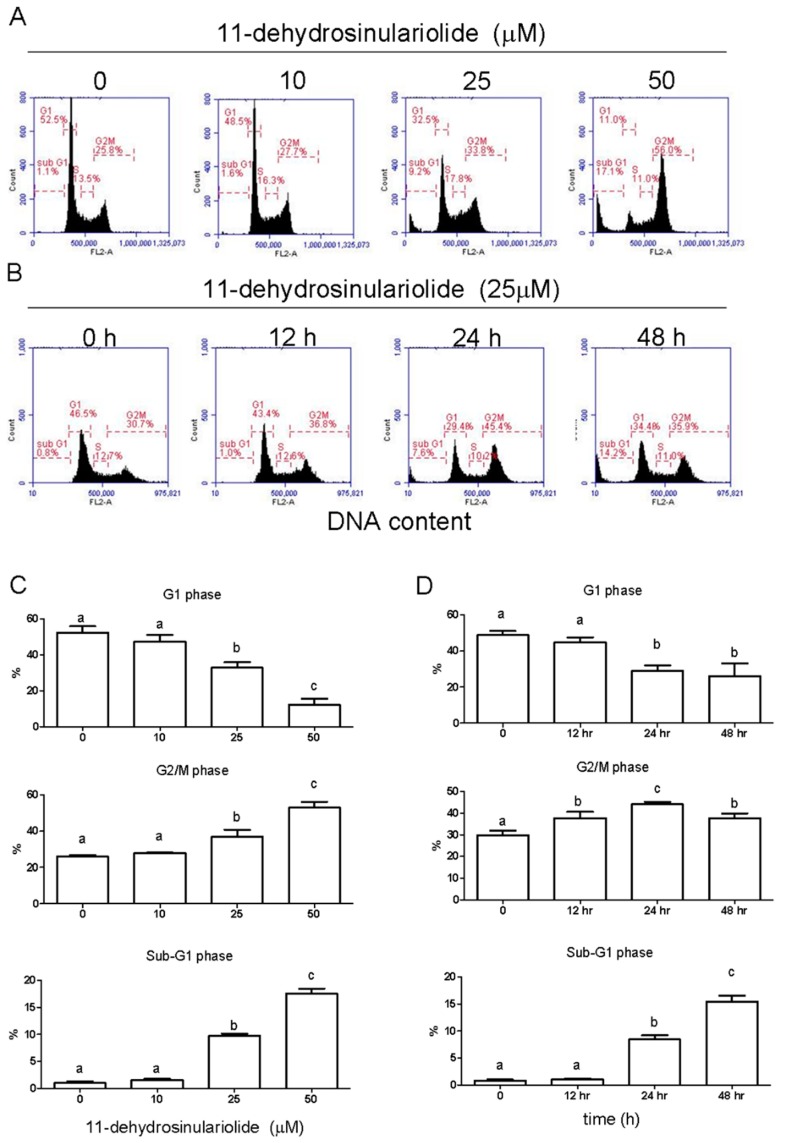
Effects of 11-dehydrosinulariolide on cell cycle progression in H1688 cells. (**A**) Distribution of H1688 cells in different stages after dose-dependent treatment with 11-dehydrosinulariolide for 24 h. and (**B**) time-dependent treatment with 25 µM 11-dehydrosinulariolide. (**C**) Percentage values of H1688 cells in the G1, G2/M and sub-G1 phases at different concentrations of 11-dehydrosinulariolide after 24 h of treatment. (**D**) Percentage values of cells in the G1, G2/M and SubG1 phases at different incubation times with 25 μM 11-dehydrosinulariolide. The data are presented as means ± SD from triplicate samples for each treatment.

**Figure 3:**
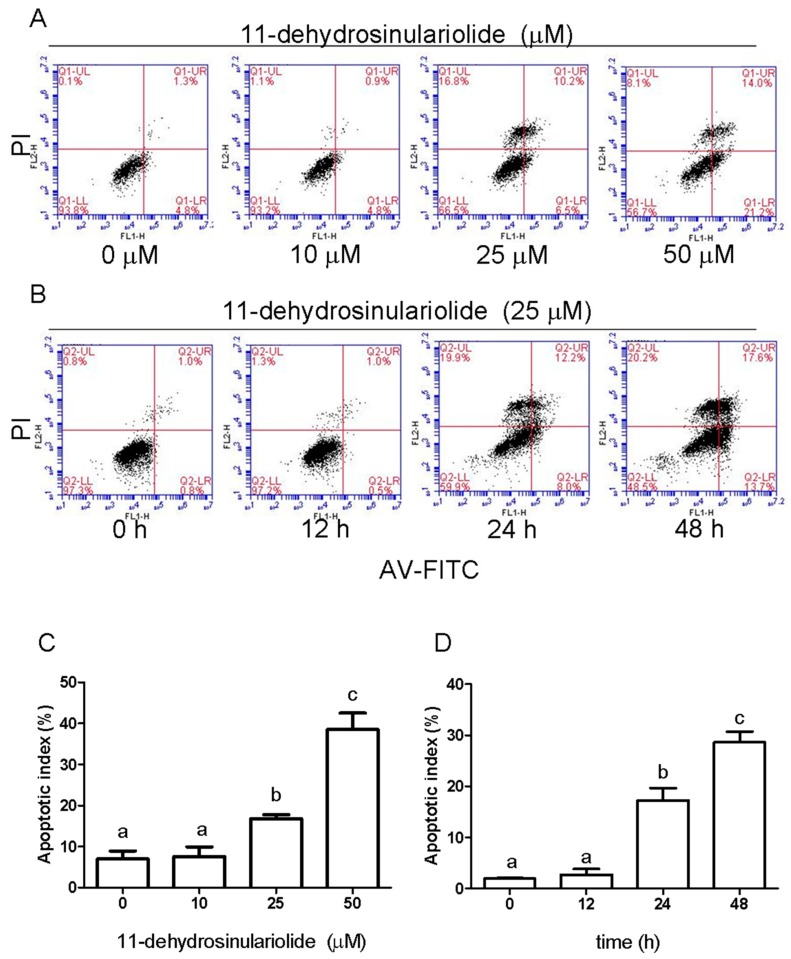
Effects of 11-dehydrosinulariolide on apoptosis in H1688 cells. (**A**) Apoptosis of H1688 cells after dose-dependent treatment with 11-dehydrosinulariolide for 24 h. and (**B**) time-dependent treatment with 25 µM 11-dehydrosinulariolide. Cell apoptosis was assessed via flow cytometry using the Annexin V-FITC apoptosis detection kit with PI (the upper left quadrant represents necrotic cells; the upper right quadrant contains late apoptotic cells; the lower left quadrant shows viable cells; and the lower right quadrant denotes early apoptotic cells). (**C**) The apoptotic index of H1688 cells at different concentrations of 11-dehydrosinulariolide after 24 h of treatment. (**D**) The apoptotic index of H1688 cells at different incubation times with 25 μM 11-dehydrosinulariolide. The data are presented as means ± SD from triplicate samples for each treatment.

To further determine whether 11-dehydrosinulariolide causes cell death by cell cycle arrest and/or apoptosis, H1688 cells were treated with 11-dehydrosinulariolide at 0, 10, 25 and 50 μM for 24 h or were treated with 25 μM 11-dehydrosinulariolide for 0, 12, 24 and 48 h. The DNA content of the cells was quantified using PI staining by flow cytometric analysis. The cell cycle comprises four different phases (G0/G1 phase, S phase, G2 phase, and mitosis), and the two checkpoints are G1/S and G2/M transitions [10]. As shown in Figure 2A,C, after treatment with 25 and 50 μM 11-dehydrosinulariolide for 24 h, the G2/M population was increased compared with that in the control condition, with a corresponding reduction in the G1 phase. In addition, Figure 2A,C shows that 25 and 50 μM 11-dehydrosinulariolide induced an increase in the number of cells in the sub-G_1_ population, which is an indication of apoptotic cell death, and these effects were dose dependent. Moreover, a time-dependent increase in cell death was observed (Figure 2B,D). To further confirm whether 11-dehydrosinulariolide causes cell death via apoptosis, H1688 cells were treated with 0, 10, 25 and 50 μM 11-dehydrosinulariolide for 24 h or were treated with 25 μM 11-dehydrosinulariolide for 0, 12, 24 and 48 h, and apoptosis was analyzed using Annexin V-FITC and propidium iodide staining and flow cytometry. As shown in Figure 3, treatment with 11-dehydrosinulariolide produced a time- and dose-dependent increase in early (Annexin V+/PI−, lower right) and late apoptotic (Annexin V+/PI+, upper right) cells but not in necrotic cells (Annexin V−/PI+, upper left). These results suggest that 11-dehydrosinulariolide induced growth-inhibitory responses through G2/M cell cycle arrest and apoptosis.

### 2.3. 11-Dehydrosinulariolide Induces H1688 Cell Apoptosis through a Caspase-Dependent Pathway

**Figure 4:**
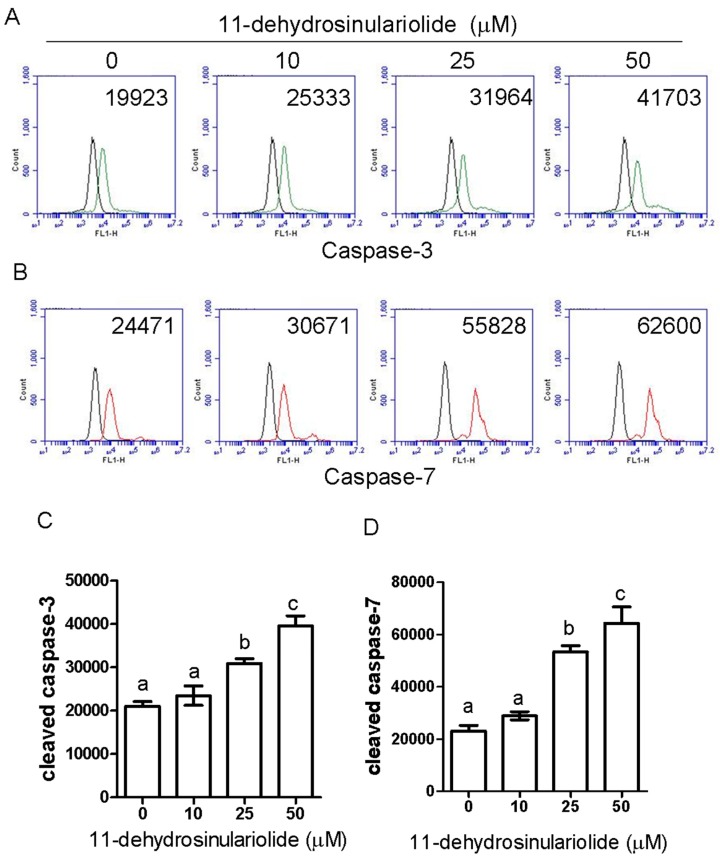

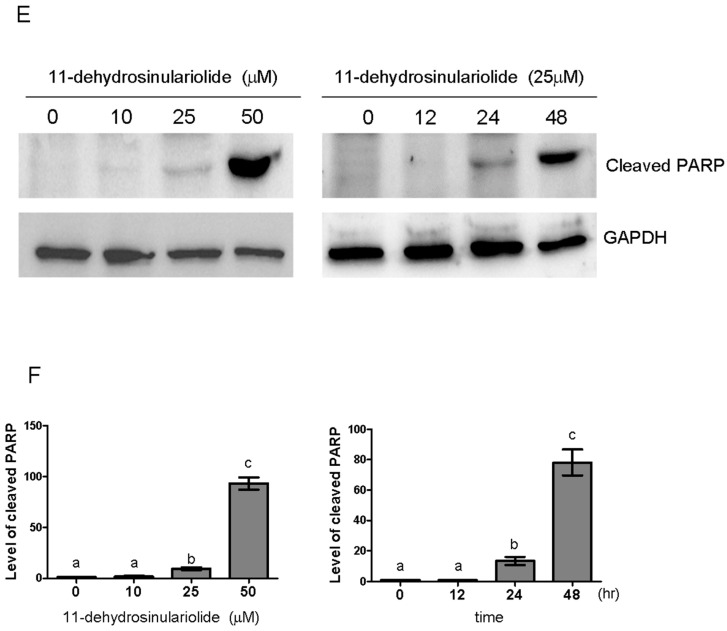
Effects of 11-dehydrosinulariolide on caspase-3 and -7 activities and cleaved-PARP in H1688 cells. H1688 cells were treated with different concentrations of 11-dehydrosinulariolide for 24 h and the activities of (**A**) caspase-3 and (**B**) caspase-7 were determined via flow cytometry. Black line: unstained H1688 cells; green line or red line: cells treated with different doses of 11-dehydrosinulariolide. (**C**,**D**) The bar data represent the means ± SD of samples from three wells. (**E**) Cell lysates of H1688 cells treated with different concentrations of 11-dehydrosinulariolide for 24 h or 25 μM 11-dehydrosinulariolide for 0, 12, 24 and 48 h were collected, and Western blotting was used to determine the cleaved-PARP protein expression level. (**F**) GAPDH was used as a loading control, and the quantified expression levels (mean ± SD) by ImageJ software were plotted in the bar graphs.

**Figure 5:**
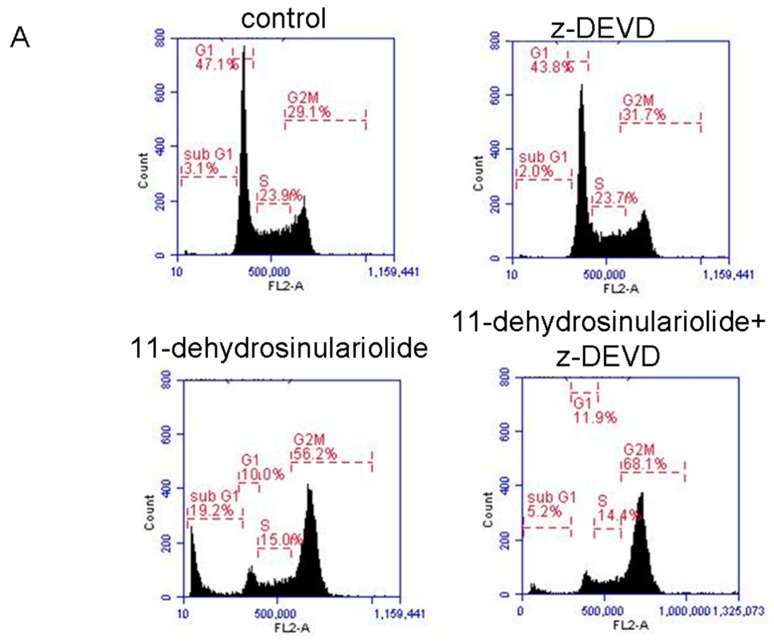

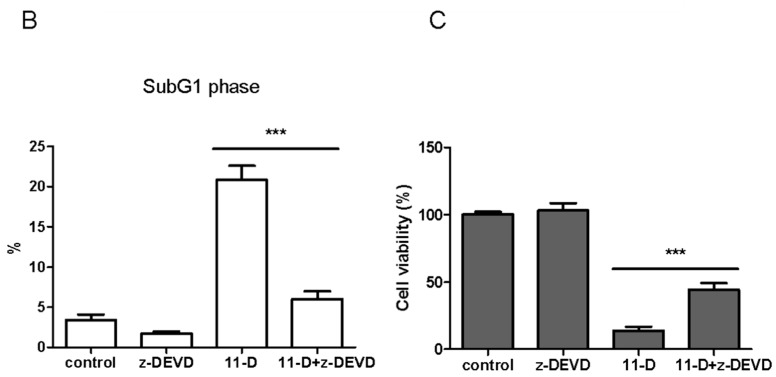

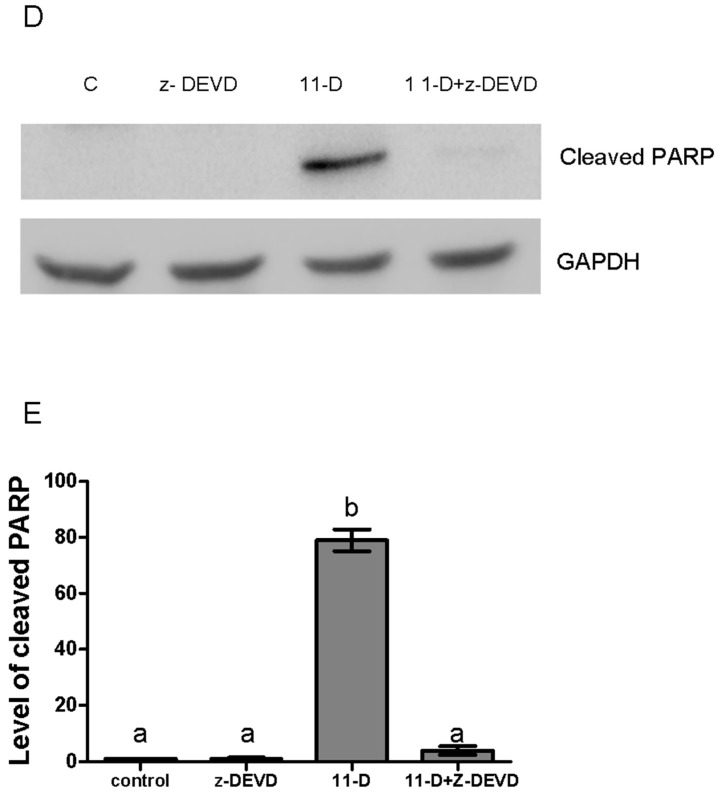
Effects of a caspase 3 inhibitor (zDEVD-fmk) on 11-dehydrosinulariolide-induced apoptosis and growth inhibition. (**A**) Distribution of H1688 cells in different stages after treatment with zDEVD-fmk and 11-dehydrosinulariolide. H1688 cells were either left untreated or were pretreated with 20 μM zDEVD-fmk for 4 h, followed by exposure to 11-dehydrosinulariolide (50 μM). After 24 h, the cells were collected and stained with propidium iodide, and the DNA content was analyzed using flow cytometry. (**B**) The bar data represent the percentage of H1688 cells in the sub-G1 phase after treatment with zDEVD-fmk and 11-dehydrosinulariolide. (**C**) The cell viability of H1688 cells after treatment with zDEVD-fmk and 11-dehydrosinulariolide (11-D). After 24 h, cell proliferation was measured using the MTT assay. (**D**) Western blotting was used to determine the cleaved-PARP protein expression level. (**E**) GAPDH was used as a loading control, and the quantified expression levels (mean ± SD) by ImageJ software were plotted in the bar graphs. The data are presented as means ± SD from triplicate samples for each treatment.

To determine whether the caspase-mediated pathway is involved in 11-dehydrosinulariolide-induced apoptosis in H1688 cells, the activities of caspase-3 and caspase-7 were determined. As shown in Figure 4, caspase-3 (Figure 4A,C) and caspase-7 (Figure 4B,D) activities in 11-dehydrosinulariolide-treated H1688 cells were increased in a dose-dependent manner. Additionally, treatment of H1688 cells with 11-dehydrosinulariolide dose- and time-dependently enhanced the cleavage of PARP (Figure 4E,F). Therefore, to further examine the effect of caspase-mediated apoptosis on the 11-dehydrosinulariolide-induced cell growth inhibition, H1688 cells were pretreated with zDEVD-fmk, an irreversible inhibitor of caspase-3, prior to 11-dehydrosinulariolide treatment, and the cell cycle and apoptosis level were analyzed. Given 50 µM 11-dehydrosinulariolide induced more significant caspase activity than 25 µM, we selected 50 µM of 11-dehydrosinulariolide for analyzing the inhibitory effect of zDEVD-fmk against caspase 3. As shown in Figure 5, treatment with zDEVD-fmk resulted in a decrease in the sub-G_1_ population (Figure 5A,B) and cleavage of PARP expression (Figure 5D,E) against 11-dehydrosinulariolide-induced apoptosis, but elevated the cell cycle arrest at the G2/M phase (Figure 5A,B). Next, we evaluated the effect of zDEVD-fmk on 11-dehydrosinulariolide-induced cell proliferation via the MTT assay. As shown in Figure 5C, pretreatment of the H1688 cells with zDEVD-fmk significantly increased the viability of the cells that were treated with 11-dehydrosinulariolide. These data suggest that caspase-mediated apoptosis contributes to 11-dehydrosinulariolide-induced growth inhibition.

### 2.4. 11-Dehydrosinulariolide Induces p53 and ATM/Chk2 Protein Phosphorylation in H1688 Cells

**Figure 6:**
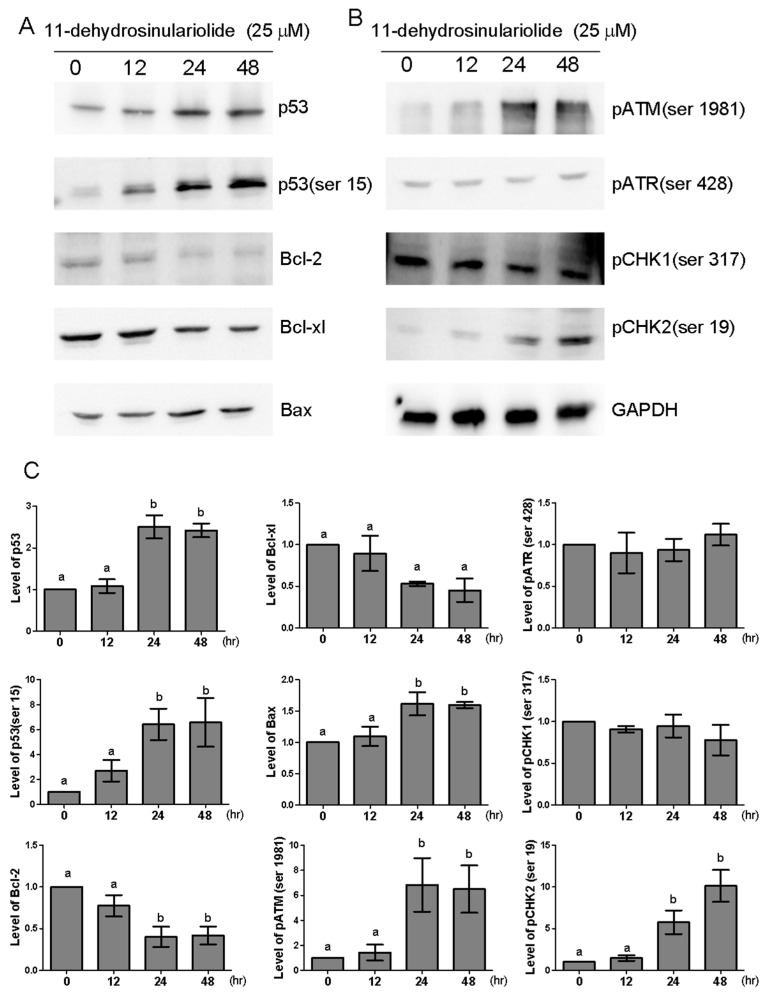
Effect of 11-dehydrosinulariolide on the expression levels of apoptosis-related proteins. Western blot analysis of proteins (**A**) p53, p53 (ser15), Bcl-2, Bcl-xl and Bax and (**B**) pATM (ser 1981), pATR (ser 428), PCHK1 (ser 317), PCHK2 (ser 19) in H1688 cells following 25-μM 11-dehydrosinulariolide treatment for 12, 24 and 48 h. Total lysates were prepared and subjected to Western blotting. (**C**) GAPDH was used as a loading control, and the quantified expression levels (mean ± SD) by ImageJ software were plotted in the bar graphs.

Previous studies have indicated that the accumulation of functional p53 plays a critical role in the chemotherapy-induced apoptosis process [11]. Western blot analysis showed a time-dependent increase in p53 protein expression at 24 and 48 h after 25-µM 11-dehydrosinulariolide treatment, accompanied by phosphorylation at Ser15 (Figure 6A,C). Additionally, DNA damage-sensing kinases, such as ATM, ATR and checkpoint kinases (Chk1 and Chk2), can control p53 protein [12,13,14,15]. The ATM/ATR pathway serves as a critical control point in DNA homologous recombination repair [16]. To examine whether DNA damage-sensing kinases are activated by 11-dehydrosinulariolide, the phosphorylation of these kinases was examined by Western blot analysis. As shown in Figure 6B,C, 25-µM 11-dehydrosinulariolide treatment significantly increased the expression of p-ATM (Ser1981) and p-Chk2 (Ser19) at 24 and 48 h. but did not affect the p-ATR (Ser428) or p-Chk1 (Ser317) levels. These data suggest that p53 might be activated via ATM or Chk2, but not via ATR or Chk1, upon 11-dehydrosinulariolide treatment.

### 2.5. 11-Dehydrosinulariolide Reduces Bcl-2 and Bcl-xl Expression and Increases Bax Protein Expression in H1688 Cells

The family of Bcl-2 proteins plays a significant role in apoptosis [17]. Additionally, previous studies have reported that 11-dehydrosinulariolide induced apoptosis through suppressing the Bcl-2/Bax ratio [8,9]. Therefore, we further examined the expression of anti-apoptosis proteins Bcl-2 and Bcl-x and the pro-apoptotic protein Bax after 25-µM 11-dehydrosinulariolide treatment. As shown in Figure 6A, 11-dehydrosinulariolide treatment in H1688 cells for 24 and 48 h. resulted in decreased Bcl-2 and Bcl-xl protein levels and increased Bax protein expression.

### 2.6. 11-Dehydrosinulariolide Upregulates PTEN and Inhibits Akt

**Figure 7:**
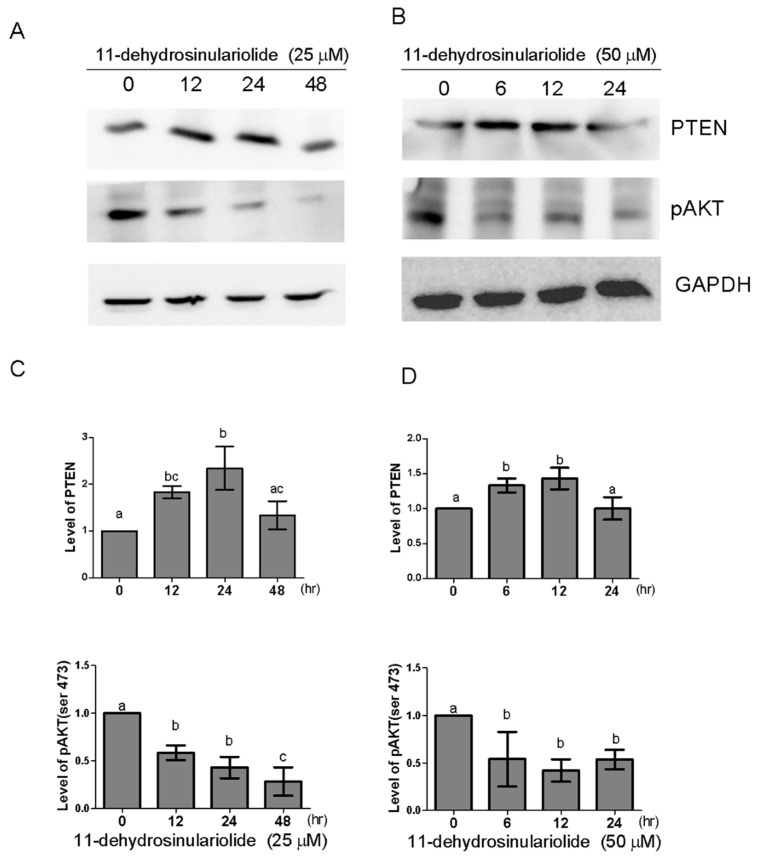
Effect of 11-dehydrosinulariolide on the expression levels of PTEN and pAKT (Ser473) proteins. H1688 cells were treated with (**A**) 25 μM 11-dehydrosinulariolide for 12, 24 and 48 h or (**B**) 50 μM 11-dehydrosinulariolide for 6, 12 and 24 h. Total lysates were prepared and subjected to Western blotting. (**C**,**D**) GAPDH was used as a loading control, and the quantified expression levels (mean ± SD) by ImageJ software were plotted in the bar graphs.

The inducible expression of the tumor suppressor gene PTEN promotes apoptosis by inhibiting the PI3K/Akt pathway [18]. To investigate whether 11-dehydrosinulariolide affects the level of PTEN in H1688 cells, the protein expression of PTEN was analyzed by Western blotting. As shown in Figure 7, PTEN was upregulated at 12 h of 25-µM or 6 h. of 50-µM 11-dehydrosinulariolide treatment, and this upregulation was sustained for up to 24 or 12 h. To determine whether the PTEN accumulation that was induced by 11-dehydrosinulariolide is functionally linked to the inhibition of AKT, the phosphorylation status of Akt was measured using a phospho-specific antibody against the Ser473 residue. The results showed that treatment with 11-dehydrosinulariolide decreased p-AKT (Ser473); therefore, PTEN accumulation may be related to the inhibition of AKT.

**Figure 8:**
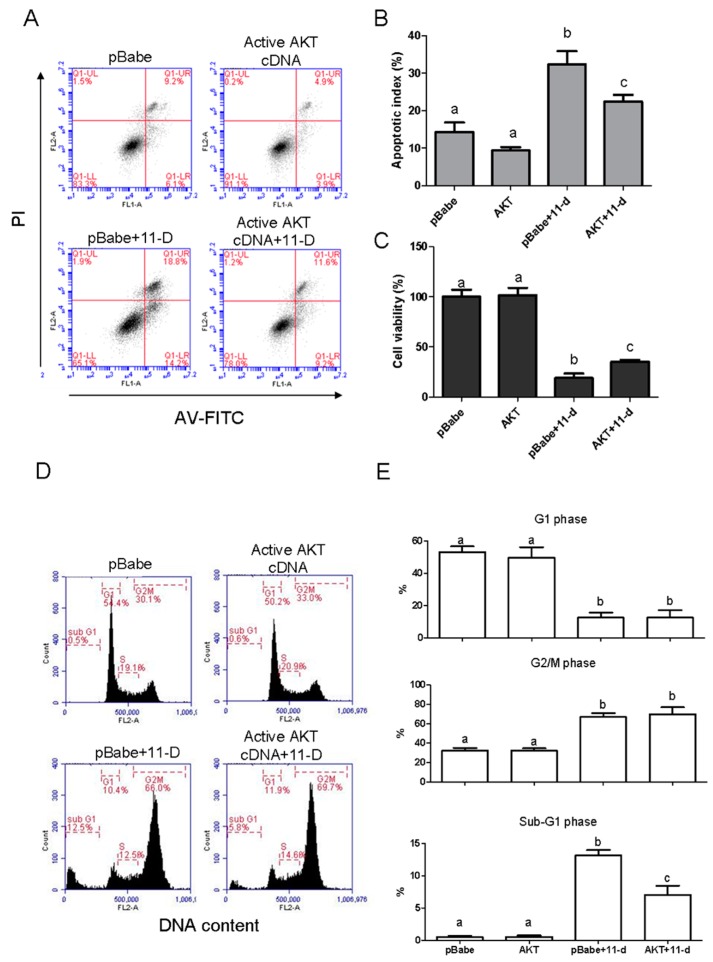
Role of AKT in 11-dehydrosinulariolide-induced apoptosis and growth inhibition in H1688 cells. Active AKT cDNA or pBabe cDNA (group) transfected cells were treated with 50 μM 11-dehydrosinulariolide for 24 h. (**A**) Apoptosis was examined by staining with annexin V-FITC/PI and was analyzed by flow cytometry. (**B**) Apoptotic index data are presented as the means ± SD from triplicate samples for each treatment. (**C**) Cell viability was evaluated by the MTT assay. (**D**) DNA histograms were constructed by PI staining and FACS flow cytometry. (**E**) The data are presented as means ± SD from triplicate samples for each treatment.

To further demonstrate that the inhibition of AKT phosphorylation is involved in 11-dehydrocaprolactone-induced growth inhibition, we transfected H1688 with a constitutively active form of active AKT cDNA. Therefore, AKT cDNA-transfected cells were then treated with 50 µM 11-dehydrolaclactone for 24 h. to analyze cell growth, cell cycle and apoptosis. As shown in Figure 8A,B, cells transfected with active AKT cDNA can reduce apoptosis induced by 11-dehydrocaprolactone, in which Annexin V-positive cells are decreased. Additionally, in a cell viability assay, AKT cDNA transfectants reduced growth inhibition by 11-dehydrosphingosine (Figure 8C). However, AKT cDNA transfectants did not affect the 11-dehydrosinulariolide-induced G2/M arrest (Figure 8D,E). Therefore, these results suggest that inhibition of AKT phosphorylation may play an important role in 11-dehydrosphingosine-induced apoptosis and growth inhibition of H1688 cells.

### 2.7. 11-Dehydrosinulariolide Induces Tumor Regression in a Mouse Xenograft Model

**Figure 9:**
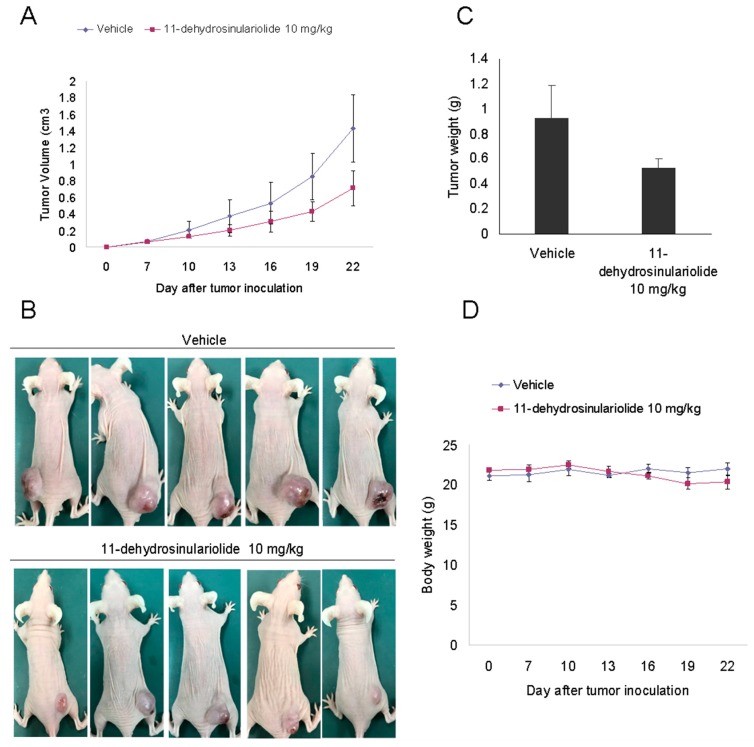
Effect of 11-dehydrosinulariolide on H1688 tumor growth in BALB/c athymic nude mice. H1688 cells (1 × 10^7^) were subcutaneously injected into the mice, and the tumors were allowed to grow to a size of approximately 80 mm^3^. Subsequently, 10 mg/kg of 11-dehydrosinulariolide was administered intraperitoneally three times a week until day 22. (**A**) Time course of tumor growth, (**B**) photograph of tumor tissues (day 22) and (**C**) the tumor weight (day 22), and (**D**) the change in the body weight were measured. The quantified data are presented as means ± SD. The data are presented as means ± SD (*n* = 5), * *p* < 0.05 compared with the vehicle control.

Finally, we examined whether 11-dehydrosinulariolide could prevent H1688 tumor xenograft progression in BALB/c athymic nude mice. As shown in Figure 9, intraperitoneal injection of 11-dehydrosinulariolide at 10 mg/kg efficiently suppressed tumor growth compared with vehicle treatment. The mean tumor volume increased from 68.2 ± 9.6 to 1413.5 ± 416.2 mm^3^ between days 7 and 22 in the vehicle control group, whereas the mean tumor volume increased from 64.5 ± 10.5 to 714.5 ± 210.9 mm^3^ in the 11-dehydrosinulariolide group (Figure 9A,B). Additionally, the tumor weights were significantly heavier in the control group than those in the 11-dehydrosinulariolide-treated group, with weights of 0.93± 0.26 g and 0.53± 0.08 g, respectively (Figure 9C).Notably, we did not observe any significant changes in mortality rate, food intake, and body weight (Figure 9D) between the 11-dehydrosinulariolide-treated and the control groups throughout the experimental period. These results suggest that 11-dehydrosinulariolide treatment significantly suppresses the growth of small lung cancer cells without a significant effect on the food intake or total body weight of the mice.

## 3. Discussion

11-Dehydrosinulariolide, a marine-derived terpenoid, has shown several bioactivities [6,7,8]. Chen et al. [6] reported that 11-dehydrosinulariolide suppressed 6-hydroxydopamine-induced cytotoxicity and apoptosis in a human neuroblastoma cell line, SH-SY5Y, and reduced the expression of inducible NO synthase (iNOS) and cyclooxygenase-2 (COX-2) proteins in lipopolysaccharide-stimulated macrophage cells. Chen et al. [7] showed that 11-dehydrosinulariolide has anti-apoptotic and anti-inflammatory effects through PI3K/Akt-dependent CREB activation and M2 microglia polarization, respectively. Liu et al. [8] reported that 11-dehydrosinulariolide induced cytotoxicity in Ca9-22 cells through both ATF6 and PERK-eIF2α-ATF4-CHOP apoptosis-inducing pathways. However, the anticancer effects of 11-dehydrosinulariolide on SCLC have yet to be evaluated. In the present study, we investigated the potential anticancer effects of 11-dehydrosinulariolide on H1688 SCLC cells and the underlying mechanisms.

Recently, increasing evidence has revealed that the dysregulation of apoptosis is related to carcinogenesis [19]. Therefore, it was pointed out that the therapeutic efficacy of chemotherapeutic agents depends on the ability of tumor cells to respond to apoptosis [20]. 11-Dehydrosinulariolide has been shown to induce caspase-dependent apoptosis in human oral squamous cell carcinoma cells [8,21] and human melanoma cells [9]. In our present study, the presence of apoptotic cells (annexin V+), activated forms of caspase-7 and caspase-3, and PARP cleavage indicated that apoptosis was involved in 11-dehydrosinulariolide-induced SCLC cell death. However, it is worth noting that in the oral carcinoma and melanoma cell lines, the concentration of 11-dehydrosinulariolide that induced apoptosis at 24 h. was 1.5–6 µg/mL (approximately 4.5–8 µM). [8,9,21] However, our study found that 10 µM 11-dehydrosinulariolide did not significantly induce apoptosis at 24 h., but a concentration above 25 µM is needed to induce apoptosis in SCLC H1688 cells. Therefore, it is important to further explore the detailed mechanism of 11-dehydrosinulariolide and explain why different cells have different effects.

Cell cycle arrest is a common cause of cell growth inhibition [22]. Unlike previous studies, our study, for the first time, found that 11-dehydrosinulariolide can induce G2/M arrest in SCLC cells. Additionally, ATM plays an important role in the activation of cell cycle checkpoints [23]. ATM is rapidly and specifically activated in response to not only this activation but also to damage induced by other cellular stresses [24,25,26]. When DNA damage occurs, activated ATM can regulate the phosphorylation status and, thus, the activity of Chk2, which subsequently induces G2/M cell cycle arrest by decreasing the protein expression of cdc25c [27]. In the present study, we first found that 11-dehydrosinulariolide activated ATM and Chk2, suggesting that the mechanisms responsible for the effects of 11-dehydrosinulariolide on G2/M phase arrest may be related to the regulation of the ATM-Chk2 signaling pathway. However, the detailed mechanism still requires more experiments to prove.

A previous study reported that ATM can phosphorylate Chk2 [28], which is involved in p53 activation [16], indicating that ATM and Chk2 are part of the pathway that leads to p53 activation. The level of p53 is controlled by the Mdm2 protein, which degrades p53 soon after synthesis [29]. When cells are subjected to certain types of genotoxic stress, ATM or Chk2 can phosphorylate p53 at multiple sites, thereby preventing Mdm2-mediated degradation [30,31,32]. Additionally, accumulation of these p53 target genes may contribute to the release of cytochrome c from the mitochondria, resulting in the activation of caspase-3 and caspase-7 by inducing the expression of proapoptotic genes, including Bax [12]. In the present study, our data showed that the expression of p53 and p53 (Ser15) was increased from 24 to 48 h of 11-dehydrosinulariolide exposure, and Bax expression was increased after 24 h of 11-dehydrosinulariolide exposure. Additionally, the levels of p-ATM (Ser1981) and p-Chk2 (Ser19) were increased during 11-dehydrosinulariolide treatment. This result parallels the rise in p-p53 (Ser15). Thus, these data suggest that 11-dehydrosinulariolide-induced apoptosis of SCLC cancer cells may be associated with the activation of the DNA damage-sensing kinases, ATM and Chk2, leading to the accumulation of p53, which, in turn, transactivates the proapoptotic Bax signaling pathway.

Bcl-2 proteins are a family of proteins involved in the response to apoptosis. Some of these proteins (such as bcl-2 and bcl-XL) are anti-apoptotic, while others (such as Bad, Bax or Bid) are pro-apoptotic and have been reported to play a pivotal role in regulating cell life and death [33]. Therefore, the balance between the anti-apoptotic and pro-apoptotic Bcl-2 family protein expression levels is important for the fate of the cells. Similar to previous results in oral carcinoma and melanoma cell lines [8,9], our data also revealed that the protein expression of antiapoptotic Bcl-2 was reduced and that of proapoptotic Bax was elevated 24 and 48 h after 11-dehydrosinulariolide treatment (Figure 6A). These results revealed the molecular events occurring during 11-dehydrosinulariolide-induced apoptosis by altering the expression of specific BCL-2 family members.

PI3K/AKT constitutes an important pathway regulating the signaling of multiple biological processes such as apoptosis, metabolism, cell proliferation and cell growth [30]. Activated PI-3K produces two second messengers, PtdIns-3,4-P2 and PtdIns-3,4,5-P3, which, in turn, phosphorylate Akt on Thr-308 and Ser-473 [31]. Activated Akt prevents apoptosis by generating antiapoptotic signals through the phosphorylation of pro-apoptotic Bcl-2 family members Bad, Bax, and caspase-9 [32,34]. PTEN is an important tumor suppressor that is frequently mutated in human cancers [35]. PTEN inhibits PI-3K/Akt signaling through the dephosphorylation of phospholipids that are produced by PI-3K [36]. In this study, a significant increase in the PTEN level was observed after 11-dehydrosinulariolide treatment. Additionally, the upregulation of PTEN was associated with a decrease in p-Akt (ser 473) levels after 12 h of 11-dehydrosinulariolide treatment. These data suggest that 11-dehydrosinulariolide-induced upregulation of PTEN is functionally associated with the inhibition of Akt and is involved in 11-dehydrosinulariolide-induced apoptosis. However, the mechanism by which 11-dehydrosinulariolide increases PTEN levels is, as yet, unknown. Given that PTEN transcription is induced by p53 [37], it is possible that PTEN is also a target of p53 upon 11-dehydrosinulariolide treatment. Further studies are required to elucidate the precise mechanism for the role of 11-dehydrosinulariolide in PTEN expression.

It is worth noting that previous research has found that the marine-derived compound 11-dehydrosinulariolide upregulates the Akt/PI3K pathway to protect SH-SY5Y human neuroblastoma cells against 6-hydroxydopamine (6-OHDA)-mediated damage [6,38]. These data oppose the results of our study. However, because we still do not know the detailed molecular mechanism evidence, we can only infer that the difference in AKT phosphorylation or dephosphorylation elicited by 11-dehydrosinulariolide might be explained by cell type differences or different stimuli.

Our results showed that the IC50 of 11-dehydrosinulariolide inhibiting cell growth in H1688 and H146 SCLC cells was approximately 29.8–435 µM and 19.1–25.1 µM at 24 and 48 h, respectively. In H1688 cells treated with 25 µM 11-dehydrosinulariolide for 24 and 48 h, apoptosis and G2/M arrest were also significantly induced. However, our colony formation assay revealed that colony formation was significantly inhibited with as high as 2.5 µM11-dehydrosinulariolide. Additionally, it was previously found that 4.5–18.04 µM 11-dehydrosinulariolide significantly inhibited the growth of OSCS and melanoma cells [8,9], as well as inhibited the metastasis of oral squamous cell carcinoma CAL-27 cells, at 1.5–6 µM [9]. Therefore, in summary, the results of the studies thus far show that 11-dehydrosinulariolide has an anticancer effect at a minimum of 1.5 μM; however, in different cell strains and different analytical methods such as cell growth and colony assays, the effective dose of 11-dehydrosinulariolide is wide ranging and the compound targets different pathways to varying degrees. Therefore, the detailed mechanism for the different effects still needs further discussion.

11-Dehydrosinulariolide is a natural compound newly discovered in 2003, but no study has investigated its therapeutic effect until 2011. Currently, it has been shown to possess anti-tumor activities only in oral carcinoma and melanoma cells [8,9,21], and our study is the first research attempted to evaluate the potential therapeutic effects in small cell lung cancer. It is difficult to affirm whether the doses of 11-dehydrosinulariolide applied in this preclinical study can be comparatively adapted to clinical dosages. However, we tested its cytotoxicity on BEAS-2B human bronchial epithelial cells (Figure 1C), and the concentration of 11-dehydrosinulariolide at 25 µM exhibited moderate but not significant cytotoxicity to BEAS-2B cells by 24 h, a dose roughly equivalent to the dosage that we used in our animal studies (10 mg/kg) where we did not observe apparent adverse effects. Hence, we believe that normal human lung cells and rodent models could be tolerant to 11-dehydrosinulariolide at such concentrations.

We have also converted the dose of 11-dehydrosinulariolide that we injected into mice to the human equivalent dose (HED), according to the formula provided by Food and Drug Administration (FDA) in the guidance for industry [39]. Presumably, the body weight of an adult human is 60 kg and that of a mouse is, on average, 0.02 kg. Thus, the HED is equal to 0.711 mg/kg, which should still be an acceptable dosage compared with that of other FDA-approved pulmonary chemotherapy agents such as nivolumab [40]. Nevertheless, more studies are required to elucidate the safety of 11-dehydrosinulariolid and to determine the minimum effective dose for future clinical trials.

In summary, our findings in this study suggest that 11-dehydrosinulariolide from soft coral is a promising precursor compound that can be used as treatment for SCLC in vivo; therefore, 11-dehydrosinulariolide deserves further research and development as an anticancer treatment.

## 4. Materials and Methods

### 4.1. Chemicals

11-Dehydrosinulariolide was isolated and purified from the cultured soft coral *Sinularia flexibilis*, as previously described [6] and was kindly provided by Jui-Hsin Su (National Museum of Marine Biology & Aquarium, Pingtung, Taiwan). Dantrolene dimethyl sulfoxide (DMSO; Sigma-Aldrich Co., St. Louis, MO, USA) was used to dissolve and dilute 11-dehydrosinulariolide to produce a 50-mM stock solution. The purity of 11-dehydrosinulariolide was 100% based on ^1^H-NMR and mass spectral analyses.

### 4.2. Cell Lines

H1688 and H146 human small cell lung cancer (SCLC) cell lines and the immortalized bronchial epithelial cell line BEAS-2B were purchased from the Food Industry Research and Development Institute (Hsinchu, Taiwan). H1688 cells are adherent cells derived from metastatic liver; however, H146 cells are suspension cells and originate from the metastatic bone marrow. These cell lines were incubated at 37 °C in a humidified atmosphere of 5% CO_2_ and were cultured in RPMI-1640 supplemented with 10% fetal bovine serum (FBS), 100 µg/mL of penicillin, and 100 μg/mL of streptomycin.

### 4.3. pBabe.puro Derived Retroviral Particle Production and Infection

pBabe.puro-Myc-Flag-PKB1/AKT, a constitutively active mutant of PKB1/AKT cloned in the retroviral expression vector was kindly provided by Chia-Che Chang (National Chung Hsing University, Taichung, Taiwan). Retrovirus was generated as previously described [41]. Briefly, HEK-293T cells (7 × 10^5^) were transiently transfected for 24 h with jetPEI™ transfection reagent (Polyplus) as indicated by the manufacturer, and 2.5 μg of pBabe-puro-based plasmids along with the plasmids producing viral particles, including gag-pol and VSV-G proteins. Viral particles released into the fresh culture media replaced at 48 h following initial transfection were harvested by centrifugation, and the supernatant containing viral particles was collected. In the virus infection test, H1688 cells (5 × 10^5^) were cultured for 48 h with virus particle medium containing 8 μg/mL of polymutation (Sigma-Aldrich, St. Louis, MO, USA); after transfection, the cells were harvested and re-inoculated onto the new culture plate for further experiments.

### 4.4. Cell Proliferation Assay

To evaluate the effect of 11-dehydrosinulariolide on H1688, H146 and Beas2B cell growth, the cells were seeded at 1 × 10^5^ cells/well in a 24-well plate overnight and were treated with 11-dehydrosinulariolide at different concentrations (0, 5, 10, 25, and 50 μM) for 12, 24 or 48 h in the absence or presence of 20 μM of an irreversible caspase 3 inhibitor (zDEVD-fmk) (BioVision, Inc., Mountain View, CA, USA). After incubation, 200 μL of 3-(4,5-dimethylthiazol-2-yl)-2,5-di-phenyltetrazolium bromide (MTT; 0.5 mg/mL; Sigma-Aldrich, St. Louis, MO, USA) was added to each well and was incubated at 37 °C for 4 h. The purple formazan crystals produced by the mitochondrial dehydrogenase enzymes were dissolved in 600 μL of DMSO and were detected at 540 nm using an ELISA microplate reader (Tecan Sunrise, San Jose, CA, USA). The concentration of 11-dehydrosinulariolide required to inhibit cell proliferation by 50% (IC50) was calculated using Microsoft Excel software for semi-log curve fitting with regression analysis.

### 4.5. Colony Formation Assay

The effect of 11-dehydrosinulariolide on SLC cancer colony formation was tested. H1688 cells were seeded at a density of 5 × 10^2^ cells/wells in 6-well plates and were cultured with 5 mL of medium for 24 h. After 24 h, the cells were treated with different concentrations (2.5, 5, 10, 25 and 50 μM) of 11-dehydrosinulariolide for 2 weeks. Next, the medium was removed, and the cells were washed twice with PBS, followed by staining for 15 min with 5 mL of Giemsa solution, rinsing with tap water, and drying at room temperature. The number of cell colonies in each well was counted. Cells treated with 0.1% DMSO were used as the control.

### 4.6. Cell Cycle Analysis

The cells were seeded at 2 × 10^5^ cells/well in a 6-well plate overnight and were incubated in culture medium containing 11-dehydrosinulariolide at different concentrations (0, 10, 25, and 50 μM) for 24 h and at 25 μM 11-dehydrosinulariolide for 0, 12, 24 and 48 h. Next, the cells were collected, washed with PBS, and fixed with 70% ice-cold ethanol at 4 °C overnight. After fixation, the cells were stained with 100 μg/mL of RNase A and 50 μg/mL of propidium iodide (PI) (Sigma-Aldrich, St. Louis, MO, USA) staining solution for 15 min, while being protected from light, and then were analyzed using an Accuri 5 flow cytometer (Accuri Cytometers; BD Biosciences, San Jose, CA, USA) equipped with C6 Accuri system software (Accuri Cytometers). All experiments were performed in triplicate and yielded similar results.

### 4.7. Annexin V/PI Staining to Detect Apoptosis

To determine whether cell death induced by 11-dehydrosinulariolide has apoptotic features, Annexin V/PI double staining was applied. Briefly, the cells were seeded at 2 × 10^5^ cells/well in a 6-well plate overnight and were treated with 11-dehydrosinulariolide at different concentrations (0, 10, 25, and 50 μM) for 24 h or with 25 μM 11-dehydrosinulariolide for 0, 12, 24 and 48 h. Apoptotic cells were determined using the Annexin V/PI binding kit (BD Biosciences, Franklin Lakes, NJ, USA) and were analyzed using an Accuri 5 flow cytometer equipped with C6 Accuri system software.

### 4.8. Caspase-3 and Caspase-7 Activities

The cells were seeded at 2 × 10^5^ cells/well in a 6-well plate overnight and were incubated in culture medium containing 11-dehydrosinulariolide at different concentrations (0, 10, 25, and 50 μM) for 24 h and were collected for the measurement of caspase-3 and -7 activities using the appropriate CaspGLOW™ Fluorescein Active Caspase Staining Kits (Biovision, Milpitas, CA, USA) according to the manufacturer’s specifications.

### 4.9. Western Blotting Analysis

The cells were seeded at 2 × 10^5^ cells/well in a 6-well plate overnight and were treated with different concentrations of 11-dehydrosinulariolide in culture medium for the indicated times and, subsequently, were lysed in RIPA Lysis Buffer (Sigma-Aldrich, St. Louis, MO, USA). After quantification using a BCA Protein Assay Kit (Thermo Fisher Scientific, Waltham, MA, USA), equal amounts of proteins (40 µg) were transferred to PVDF membranes and were separated (Merck Millipore, Billerica, MA, USA). The membranes were incubated with the appropriate primary antibody overnight at 4 °C, followed by a 1-h incubation with horseradish peroxidase (HRP)-conjugated goat anti-rabbit IgG (H + L) secondary antibodies (cat. no. 111-035-114; Jackson ImmunoResearch Laboratories, West Grove, PA, USA). Proteins were detected using an ECL Western Blotting Substrate Kit (ECL prime Western Blotting Substrate; GE Healthcare Life Sciences, Piscataway, NJ, USA) and were analyzed using the Hansor Luminescence Image system (Taichung, Taiwan). All bands in the blots were normalized to the level of GAPDH for each lane. The band density was measured using the ImageJ v1.47 program for Windows from the National Institutes of Health (NIH) (Bethesda, MD, USA). The primary antibodies-targeting cleaved RARP (clone 46D11; cat no. 9352; 1:1000), p53 (clone 7F5; cat no. 2527; 1:1000), p53 (Ser15) (polyclonal; cat no. 9284; 1:1000), Bax (D2E11; cat no. 5023; 1:1000), Bcl-2 (D55G8; cat no. 4223; 1:1000), Bcl-xl (54H6; cat no. 2764; 1:1000), pATM (Ser1981) (clone D25E5; cat. no. 13050; 1:500), pATR (Ser428) (polyclonal; cat no. 2853; 1:1000), pChk1 (Ser317) (clone D12H3; cat. no. 12302; 1:1000), pChk2 (Ser19) (polyclonal; cat no. 2666; 1:500), PTEN (clone D4.3; cat. no. 9188; 1:1000), pAKT (Ser473) (clone D9E; cat. no. 4060; 1:500), and GAPDH (clone D16H11; cat. no. 5174; 1:2000) were purchased from Cell Signaling Technology (Danvers, MA, USA).

### 4.10. In Vivo Antitumor Experiment

Female BALB/c 6-week BALB/c athymic nude mice (weight, 20 ± 2 g) were obtained from the National Laboratory Animal Center (Taipei, Taiwan) and were raised at the National Chung Hsing University (NCHU) Animal Experiment Research Center. The mice were fed sterilized mouse chow and water. Laboratory animal management was performed by the Laboratory Animal Management and Ethics Committee (IACUC no. 107-127) of NCHU. The mice were randomly divided into the following two groups (*n* = 5): vehicle control (10% DMSO + 90% glyceryl trioctanoate (Sigma-Aldrich, St. Louis, MO, USA), three times weekly, intraperitoneal (ip) injection) and 11-dehydrosinulariolide (10 mg/kg, three times weekly, ip injection). According to the guidance for industry from the Food and Drug Administration (FDA) [39], the human equivalent dose (HED) can be calculated simply using the following Formula:

HED (mg/kg) = Animal dose (mg/kg) × (Animal Weight (kg)/Human weight (kg))^0.33^

In this study, we used the dose of 10 mg/kg for mice and a mouse weight = 0.02 kg and assumed a human weight of 60 kg. The HED = 0.711 mg/kg.

HED (mg/kg) = 10 (mg/kg) × (0.02 (kg)/60 (kg))^0.33^

In total, 1 × 10^7^ H1688 cells in 200 μL of extracellular matrix gel were transplanted into the back area under the skin of the animal. Treatment was initiated on the 7th day after transplantation (the tumor cell size is less than 80 mm^3^). During the whole experimental period, the feed intake and motor activity of the mice were carefully observed, their body weights were measured, the tumor volumes were measured three times a week using an electronic caliper, and the tumor volume was calculated according to the following formula: a × b^2^ × 0.5, where a indicates the tumor’s long diameter, and b indicates the tumor’s short diameter. At the end of the study (day 22), the mice were sacrificed, the tumors were excised carefully, and the tumor weights were measured.

### 4.11. Statistical Analysis

The data were expressed as means ± SD. GraphPad Prism 5 (GraphPad Software Inc., San Diego, CA, USA) was used to analyze the significance of the differences among the groups. One-way ANOVA followed by Tukey’s posttest was performed for comparisons among groups, and unpaired two-tailed *t* test was performed to analyze the differences between two independent groups. *p* < 0.05 was considered statistically significant. Similar results were obtained from three independent experiments.
